# Evidence for nuclear interaction of a cytoskeleton protein (OsIFL) with metallothionein and its role in salinity stress tolerance

**DOI:** 10.1038/srep34762

**Published:** 2016-10-06

**Authors:** Neelam Soda, Ashutosh Sharan, Brijesh K. Gupta, Sneh L. Singla-Pareek, Ashwani Pareek

**Affiliations:** 1 Stress Physiology and Molecular Biology Laboratory, School of Life Sciences, Jawaharlal Nehru University, New Delhi 110067, India; 2 Plant Stress Biology, International Centre for Genetic Engineering and Biotechnology, Aruna Asaf Ali Road, New Delhi 110067, India

## Abstract

Soil salinity is being perceived as a major threat to agriculture. Plant breeders and molecular biologist are putting their best efforts to raise salt-tolerant crops. The discovery of the *Saltol* QTL, a major QTL localized on chromosome I, responsible for salt tolerance at seedling stage in rice has given new hopes for raising salinity tolerant rice genotypes. In the present study, we have functionally characterized a *Saltol* QTL localized cytoskeletal protein, intermediate filament like protein (*OsIFL*), of rice. Studies related to intermediate filaments are emerging in plants, especially with respect to their involvement in abiotic stress response. Our investigations clearly establish that the heterologous expression of OsIFL in three diverse organisms (bacteria, yeast and tobacco) provides survival advantage towards diverse abiotic stresses. Screening of rice cDNA library revealed OsIFL to be strongly interacting with metallothionein protein. Bimolecular fluorescence complementation assay further confirmed this interaction to be occurring inside the nucleus. Overexpression of OsIFL in transgenic tobacco plants conferred salinity stress tolerance by maintaining favourable K^+^/Na^+^ ratio and thus showed protection from salinity stress induced ion toxicity. This study provides the first evidence for the involvement of a cytoskeletal protein in salinity stress tolerance in diverse organisms.

Abiotic stresses pose a major threat to agriculture worldwide. At the same time, it is also known that these stresses affect the yield, which mostly depends on the genetic makeup of the crops^1^,^2^,^3^. Salinity stress is one of the most detrimental factors for crop growth and production in arid regions. It has been estimated that by 2050, 50% of the arable land would be salinized and hence will be rendered unproductive^4^. In India, more than 20 Mha of land is already affected by salinity^5^. As an effort to survive under these unfavourable conditions, plants reprogram various molecular, biochemical and physiological pathways within their cells. Thorough understanding of these adaptive responses in plants, especially employing the contrasting genotypes and using the tools of functional genomics is must to achieve the target of raising salinity stress tolerant crop plants. The problem of salinity is characterized by an excess of inorganic salts, especially sodium. Climatic conditions in arid and semi arid regions lead to higher rates of evapotranspiration, resulting in a natural increase in salt concentration in soil. This increased concentration of soluble salts affect plant growth by restricting water uptake through roots. High concentrations of salts interfere with the process of absorption of essential nutritional ions by plants^6^. Increased salinity leads to physiological and metabolic disturbances in plants, affecting several vital processes such as seed germination, survival, development, photosynthesis, respiration and ultimately, the yield^7^.

Though rice is rated as a salt-sensitive crop^8^,^9^, some degree of varietal differences exists in their salt tolerance. Susceptibility of rice plants to salinity stress varies with their growth stage. Several studies have established that rice is extremely sensitive to salinity at germination, seedling growth and reproductive stage^10^,^11^. Recent research from our laboratory has documented the usefulness of exploiting the contrasting genotypes towards deciphering the physiological and molecular basis of salinity tolerance in rice at the seedling stage^12^,^13^,^14^,^15^,^16^,^17^,^18^. As a salinity tolerant check, we have employed Pokkali, a salt tolerant but low yielding landrace, while IR64, a high yielding genotype of rice has been used as a salt sensitive check in our studies. These efforts have resulted in mining of a few novel genes, some of which have been validated for their roles in stress tolerance by using functional genomics tools^16^,^17^,^18^. As salinity tolerance is a quantitative trait, understanding the physiological mechanisms of salt responses in plants, their biochemical basis and inheritance is a daunting, but an essential task, to be achieved for developing salt tolerant high yielding rice genotypes. In one of the recent reports, we have carried out comparative expression profiling of signaling related genes localized within the *Saltol*, a major salinity related QTL of rice^15^. We have shown differential regulation of several stress related genes, including intermediate filament encoding gene, in the contrasting genotypes of rice under salinity stress^15^. Several research groups have suggested participation of cytoskeletal proteins, such as actin and myosin in plant stress responses^19^ but, not much is known regarding intermediate filaments and their role in stress tolerance. The gene cloned and characterized in the present study has been annotated as intermediate filament encoding gene in Rice Genome Annotation Project (http://rice.plantbiology.msu.edu/cgi-bin/ORF_infopage.cgi?orf=LOC_Os01g18840), but we have referred to this protein as intermediate filament like protein (OsIFL). The objectives of the current study were to (1) isolate the full length gene encoding OsIFL, from *O. sativa* cv IR64 (2) test, whether the overexpression of OsIFL improves salinity tolerance in model systems such as bacteria, yeast and tobacco, (3) find out the interacting partners of OsIFL protein and finally, (4) comment on possible mechanism responsible for OsIFL-mediated stress response.

## Results

### Isolation and characterization of an intermediate filament like gene (*OsIFL*) from *Oryza sativa L*

Rice genome contains two genes for intermediate filaments like proteins (OsIFL), LOC_Os01g18840 (genomic sequence length: 1562 nucleotide; CDS length: 807 nucleotide, protein length: 269aa) localized within *Saltol* QTL of chromosome 1 and LOC_Os05g04530 (genomic sequence length: 4205 nucleotide; CDS length: 1035 nucleotide, protein length: 345aa) localized on chromosome 5. In the present study, we focused our study on chromosome 1 localized *OsIFL* (LOC_Os01g18840), since it is residing within *Saltol* QTL of rice. Secondary structure prediction of OsIFL suggested presence of 5 alpha helices, a characteristic feature of intermediate filament proteins (Figure S1a). This gene has been annotated as intermediate filament encoding gene in Rice Genome Annotation Project with multicoil structure and significant homology with IF like proteins from various other plant species (http://rice.plantbiology.msu.edu/cgi-bin/ORF_infopage.cgi?orf=LOC_Os01g18840). Further study of OsIFL, using MultiCoil software^20^, also predicted a coiled coil motif at its N-terminus (Figure S1b), thus confirming it to be a true ‘plant intermediate filament protein’.

### OsIFL is localized in cytosol as well as in nucleus

**Figure 1:**
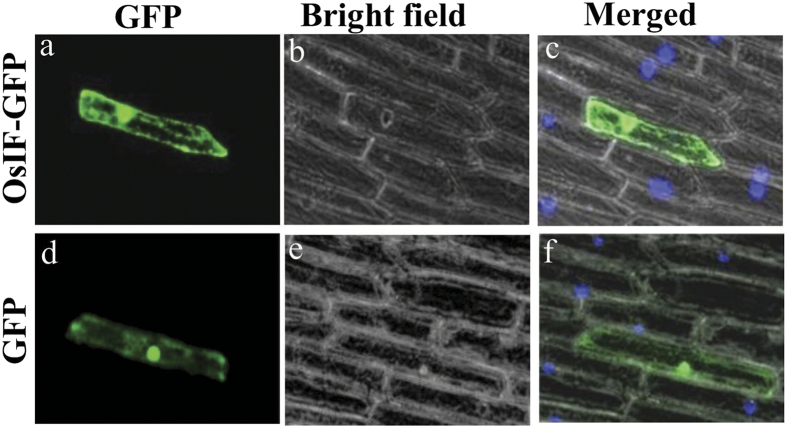
OsIFL is expressed in nucleus, cytoplasm and cell margins of plant cells. OsIFL-GFP fusion protein was transiently expressed in onion epidermal peel cells and visualized using a laser scanning confocal microscope. (**a**) OsIFL-GFP localization using confocal microscopy in onion peel epidermal cells reveals it to be forming an intracellular fibrillar network in the cytoplasm. OsIFL-GFP fusion protein fluorescence was also observed at the cell margins as well as nucleus; (**d**) GFP localization as a control; (**b**,**e**) bright field image of the corresponding onion peel; (**c,f**) Overlapping images from bright field, DAPI and GFP fluorescence.

Onion epidermal peel cells bombarded with the vector carrying OsIFL-GFP showed its expression in the form of a fibrillar network throughout the cytoplasm as evident from Fig. 1a. High expression of OsIFL-GFP protein was also observed in the nucleus and cell margins thus indicating the presence of OsIFL protein throughout the cell. Similar pattern of fluorescence was observed in most of the bombarded cells. On the other hand, onion epidermal peel cells transformed with the vector carrying only the full-length cDNA corresponding to GFP, showed dispersed GFP fluorescence in the whole cell (Fig. 1d).

### Ectopic expression of OsIFL leads to improved tolerance to multiple abiotic stresses in bacterial cells

**Figure 2:**
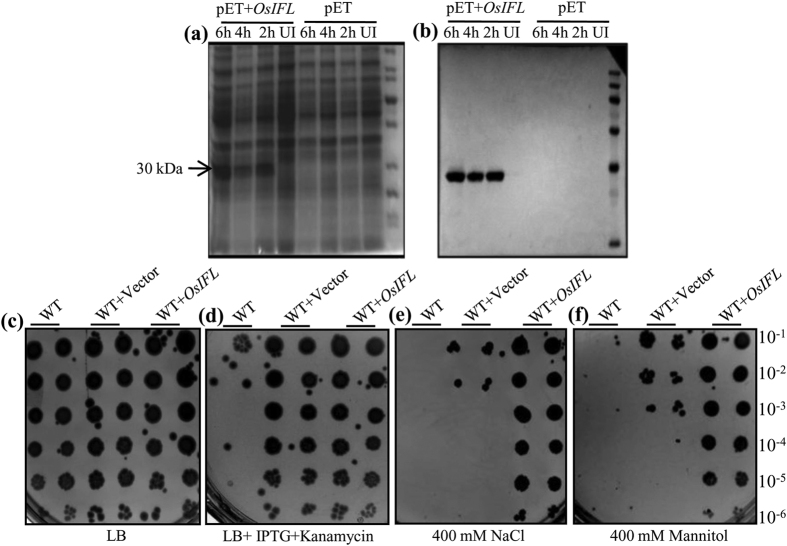
Heterologous expression of OsIFL in *E. coli* cells improves their tolerance to salinity and osmotic stress. (**a**) Induction of OsIFL protein expression by 0.3mM IPTG at 22 °C, UI: un-induced, 2h, 4h and 6h: hours after IPTG induction. pET: proteins extracted from vector alone transformed cells. pET+*OsIFL*: proteins extracted from pET+*OsIFL* transformed cells; (**b**) Western blotting using anti-His antibodies. Relative growth of WT cells (BL21), vector transformed (WT+ vector) and pET*OsIFL* transformed cells (WT+*OsIFL*) on (**c**) LB media; (**d**) LB supplemented with 0.3 mM IPTG and kanamycin; (**e**) LB supplemented with 0.3 mM IPTG, kanamycin and NaCl (400 mM) or (**f**) Mannitol (400 mM). Initial O.D_600_ ~ 0.3 and subsequent serial dilution (10^−1^ to 10^−6^) used for these assays. Plates were incubated at 37 °C and images were taken after 72 h.

To investigate the role of OsIFL in abiotic stress response, *OsIFL* cDNA was ectopically expressed in *E. coli* (BL21) cells using pET28a expression vector (Fig. 2). Coomassie stained gels of total protein extracts from pET28a*OsIFL* harbouring bacterial cells showed an extra band of 30 kDa, which was absent in pET28a (vector control) harbouring bacterial cells after IPTG induction (Fig. 2a). This 30 kDa band was detected as OsIFL-His fusion protein in western blots developed using anti-His antibodies (Fig. 2b). Peptide sequencing using MALDI-TOF-TOF further confirmed the identity of the induced protein as OsIFL corresponding to LOC_Os01g18840. After confirmation of induction of recombinant OsIFL protein in bacterial cells, these were used for a quick assay of serial dilution on LB media plates containing different stressors, such as NaCl or Mannitol, along with untransformed cells and vector alone transformed cells. While all type of bacterial cells (untransformed cells i.e. WT, WT+Vector alone and WT+*OsIFL*) could grow on LB (Fig. 2c), only cells transformed with either vector alone or vector containing the gene construct could grow in presence of kanamycin (Fig. 2d). In the presence of salinity stress (400 mM NaCl), cells expressing OsIFL showed better growth and survival in comparison to those cells transformed with only the empty vector. Where WT+*OsIFL* transformed cells could grow properly in presence of 400 mM NaCl at all the dilutions, no colonies could be detected for vector alone transformed cells after 10^−2^ dilution (Fig. 2e). *OsIFL* transformed BL21 cells also showed higher tolerance (in terms of growth) towards osmotic stress i.e. in the presence of 400 mM mannitol in media plates than the vector transformed BL21 cells (Fig. 2f). Taken together, these results clearly indicate that OsIFL plays a key role in salinity and osmotic stress tolerance in the bacterial system.

### OsIFL protein expression in *S. cerevisiae* cells protects them from the damage caused by extreme osmotic stress conditions

**Figure 3:**
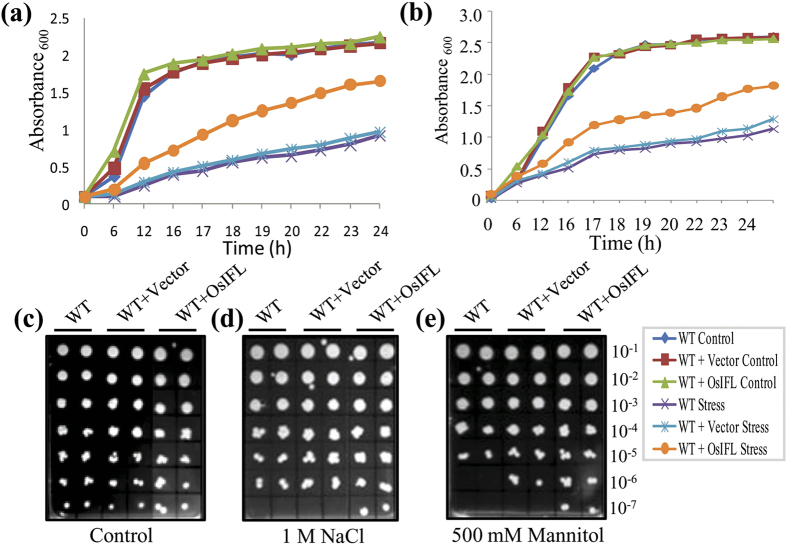
Heterologous expression of OsIFL in yeast cells improves their tolerance to salinity and osmotic stress. Growth curve assay of WT, vector transformed (WT+vector) and pYES2*OsIFL* transformed (WT+*OsIFL*), BY4741 *S. cerevisiae* cells in the presence of (**a**) 1M NaCl and (**b**) 1M Mannitol showed vigorous growth of pYES2*OsIFL* transformed cells in comparison with WT and vector transformed cells. Growth of these strains observed on (**c**) Solid YPG media; (**d**) Solid YPG media containing 1M NaCl or (**e**) 500 mM Mannitol. Initial O.D_600_ ~ 0.3 and subsequent serial dilution (10^−1^ to 10^−7^) used for these assays. Plates were incubated at 30 °C and images were taken after 72 h. WT - untransformed cells (WT), WT+ vector - vector transformed cells and WT+*OsIFL* - pYES2*OsIFL* transformed cells.

Since heterologous expression of OsIFL in *E. coli* cells enhanced their tolerance towards stress conditions, we decided to extend our studies to unicellular non-photosynthetic eukaryote - *S. cerevisiae*. *OsIFL* was cloned into yeast expression vector pYES2 under a galactose-inducible GAL1 promoter, generating pYES2OsIFL. The wild-type (WT) yeast cells (strain BY4741) were transformed with pYES2OsIFL or pYES2 vector alone and subsequently selected on SD (-ura) medium. Subsequently, all types of yeast cells were grown on YPG media containing 2% galactose. Growth of transformants (pYES2 vector or pYES2 vector carrying *OsIFL* cDNA under a galactose-inducible promoter) and WT yeast cells were compared in liquid YPG media (control), YPG media supplemented with 1M NaCl and 1M Mannitol, for 24 h. While growth of WT cells or those containing WT+vector or WT+*OsIFL* were comparable under non-stress conditions (control), these transformants exhibited a contrasting growth behavior in the presence of both salinity (1M NaCl) and osmotic stress (1M Mannitol) in liquid media. Cells expressing OsIFL (WT+*OsIFL*) grew more vigorously as compared to the WT cells or even the vector transformed cells (WT+vector) in the presence of salt and mannitol (Fig. 3a,b). Growth of these yeast cells was also assessed by serial dilution assay on solid YPG media (as shown in Fig. 3c–e), supplemented with either 1 M NaCl or 500 mM mannitol. All these yeast cells grew well on YPG media (Fig. 3c), while in the presence of stressors (NaCl or mannitol) in the media, pYES2*OsIFL* transformed cells showed better growth in comparison to WT or vector transformed cells (Fig. 3d,e). These results clearly establish a key role for OsIFL towards regulating stress responses in lower organisms, where presence of OsIFL protects cells from the osmotic stress-induced damage.

### Overexpression of OsIFL protects transgenic tobacco plants from salinity-induced damage by retaining higher levels of chlorophyll and maintaining better ion homeostasis

To investigate the relationship between OsIFL protein levels *vis a vis* stress responsiveness in higher plants, this protein was overexpressed in transgenic tobacco. Hygromycin-resistant transgenic tobacco lines overexpressing OsIFL were confirmed by tissue PCR and RT-PCR using different primer combinations (Figure S2). Among the 25 putative transgenic lines screened, 9 showed the desired amplicon of ~849 bp (a few representative lines are shown in Figure S2a) thus confirming their transgenic nature. Out of the confirmed transgenic lines, two representative lines viz. L2 and L3 were chosen for further detailed investigations. Expression of transgene was studied by RT-PCR, showing the presence of higher levels of *OsIFL* transcripts in both the L2 and L3 lines, which was absent in WT plants (Figure S2b). In Southern blot analysis, a single band corresponding to *OsIFL* was visible in both these lines, confirming integration of the transgene into the tobacco genome (Figure S2c). Lane 1 (WT tobacco) did not show any cross hybridizing band, as probe was highly specific to rice *OsIFL* gene. Western blot analysis (using anti-IF antibodies) of total protein extract from these lines showed expression of OsIFL protein in transgenic tobacco lines (Figure S2d). Lane 1 and 2 (proteins from WT tobacco under non stress and salinity stressed conditions, respectively) did not show any corresponding band suggesting high specificity of anti-IF antibodies for rice protein. It was observed here that the transgenic line L3 showed protein amount for OsIFL higher than the L2 line. As we have used 35S constitutive promoter to drive the expression of transgene in transgenic tobacco, OsIFL protein was not found to be further induced by salinity stress in any of these lines.

**Figure 4:**
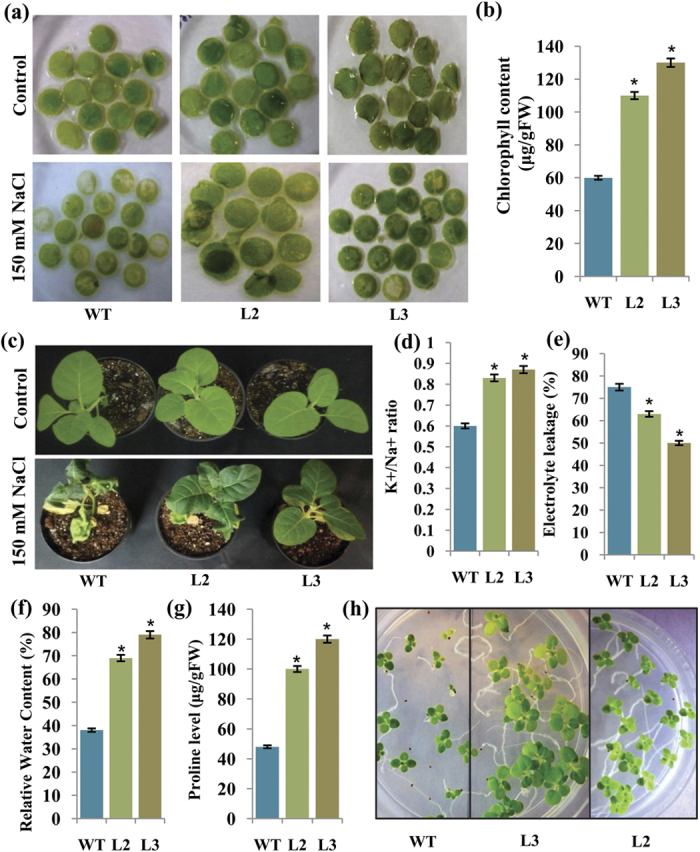
Overexpression of OsIFL in transgenic tobacco improves its tolerance to salinity stress by maintaining chlorophyll content and favorable K^+^/Na^+^ ratio. (**a**) Leaf discs of tobacco (WT, L2 and L3) after 48 hr of incubation in 150 mM NaCl (bottom row) and their corresponding control kept in half MS alone (upper row); (**b**) Bar graph showing chlorophyll content in leaf discs of tobacco plants obtained from salinity stressed tissue; (**c**) Pot assay for assessing relative growth of WT and transgenic plants (T_1_ generation) in response to salinity stress (150 mM NaCl, 3 weeks); (**d**) K^+^/Na^+^ ratio; (**e**) Electrolyte leakage (%); (**f**) Relative water content (%); (**g**) Estimated proline levels; (**h**) Germination assay of T_2_ generation seeds of tobacco plants overexpressing OsIFL in comparison to WT tobacco seeds in the presence of 150 mM NaCl. Equal numbers of wild type and OsIFL seeds (Line L2 and L3) were inoculated on MS supplemented with 150 mM NaCl; OsIFL tobacco seeds show higher germination rate as well as better seedling growth as assessed visually. Experiments were repeated thrice and s.d. values are shown as error bars. The asterisk indicates a significant difference from the wild type (WT) groups (p < 0.05, student’s t-test).

Transgenic plants overexpressing OsIFL matured normally under optimum growth conditions (28 °C, 12h L/D). To have a quick assessment of salinity tolerance in transgenic plants, leaf disc senescence assay for chlorophyll retention was performed in the presence of 150 mM NaCl. Within 48 h of stress treatment, significant difference in the “greenness” of the leaf discs was observed between WT and transgenic plants. As compared to WT, both L2 and L3 transgenic lines showed significantly enhanced tolerance towards salinity (Fig. 4a). In presence of salinity stress, line L2 and line L3 showed higher content of chlorophyll (almost double) as compared to the WT plants. In this assay, the line L3 outperformed line L2 (Fig. 4b), which is in direct correlation with higher expression of OsIFL protein in the former (Figure S1d).

To assess the relative tolerance at the whole plant level, pot assay was carried out using the T_1_ generation tobacco plants. For this purpose, the 30 day old plants (WT, Line L2 and L3) were used and one set of these plants was subjected to salinity stress (150 mM NaCl) while the other set was continued to be watered normally as controls. The overall growth of WT and transgenic plants was compared and no morphological differences were observed under control conditions. However, after three weeks of salinity stress, the WT plants showed stress induced yellowing and drooping of leaves, while this injury was observed to be minimal in OsIFL transgenic plants (Fig. 4c). To estimate the extent of damage, various biochemical and physiological parameters were analyzed in the WT and transgenic plants under stress conditions. Transgenic seedlings outperformed WT seedlings in terms of K^+^/Na^+^ ratio, electrolyte leakage, relative water content (RWC), proline content, under salinity stress (Fig. 4d–g). Measurement of Na^+^ and K^+^ concentration revealed K^+^/Na^+^ ratio to be ~40% higher in transgenic lines than the WT (Fig. 4d). These observations suggested that the *OsIFL* expressing tobacco seedlings were able to maintain K^+^/Na^+^ homeostasis better than WT plants under salinity stress. Transgenic seedlings were also capable of maintaining their membrane integrity than WT seedlings as electrolyte leakage of WT seedlings was higher than transgenic seedlings (Fig. 4e) under similar conditions. Further, WT seedlings showed drastic reduction (~60%) in relative water content (RWC) in the presence of salt stress, whereas transgenic seedlings maintained their RWC with only slight reduction (25% in L2 and 20% in L3) under stress conditions (Fig. 4f). Similarly, accumulation of proline was more than double in transgenic seedlings than WT plants, in the presence of salinity stress (Fig. 4g).

To check for the genetic and functional stability of the introduced trait, seed germination assay of tobacco seeds (T_2_ generation) overexpressing OsIFL was carried out in presence of salinity stress. Equal number of seeds (20 each) of transgenic lines (L2 and L3) and WT tobacco plants were inoculated on MS media supplemented with 150 mM NaCl. After 15 days, differential germination response of WT and transgenic seeds towards salinity stress was observed (Fig. 4h). In the presence of 150 mM NaCl, WT seeds exhibited low percentage (60%) of seed germination in comparison to transgenic seeds, where seed germination was almost 100%. WT seeds also showed delayed germination and reduced growth of seedlings than the OsIFL (L2 and L3) transgenic lines in the presence of salt stress (Fig. 4h).

### Stress marker genes are differentially regulated in OsIFL overexpressing plants under both non-stress and salinity stress conditions

**Figure 5:**
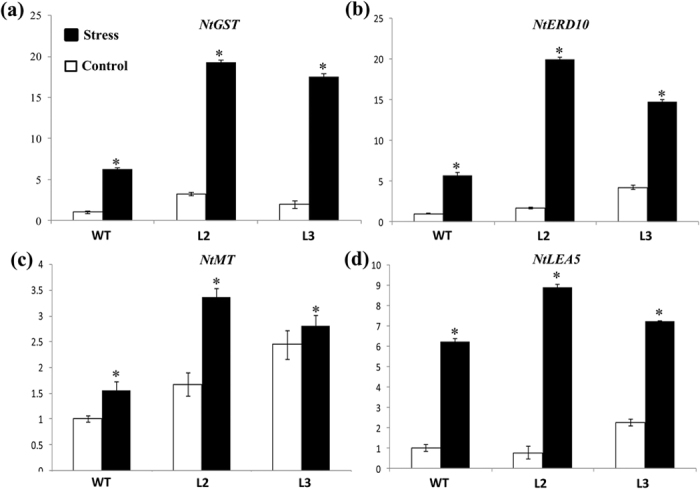
Tobacco plants overexpressing OsIFL show high constitutive expression and strong induction of stress marker genes in response to salinity stress. Four-week-old tobacco seedlings were transferred to half MS media alone (control) or media containing 200 mM NaCl (salt stress) for 24 h. Leaf tissues were used to examine the qRT-PCR based expression of (**a**) *NtGST;* (**b**) *NtERD10;* (**c**) *NtMT* and (**d**) *NtLEA5* genes. n = 3 and s.d. is shown as bar on each observation. The asterisk indicates a significant difference between control and stress samples (p < 0.05, student’s t-test).

Expression studies of stress related genes are being used to analyze the state of stress responsive pathways in plants^21^. We studied transcript abundance of four key stress marker genes, *viz*. glutathione S-transferase (GST), early-responsive to dehydration stress (ERD10), metallothionein (MT) and late embryogenesis abundant protein (LEA), in the wild type and transgenic tobacco plants by qRT-PCR. Transcript abundance of three marker genes *viz*. ERD, LEA and GST were detected to be low in WT plants and relatively high in transgenic lines under non-stress conditions (Fig. 5a,b,d). However, transcripts of these genes got induced in both wild type and transgenic plants in response to 200 mM NaCl salinity (Fig. 5a,b,d), but the level of induction was very high in the latter. Transcripts for MT were also induced by salinity stress in transgenic plants to levels higher than WT (Fig. 5c). These results suggested that OsIFL may contribute in salt stress response by regulating expression of these and many other stress-related genes.

### OsIFL interacts with several key stress-responsive proteins such as OsMT, plasma membrane ATPase, palmitoyltransferase TIP1 and proton pump interactor

Study of interacting partners of a given protein may contribute towards assigning it a probable role. For identifying the interacting partners of OsIFL, rice cDNA library in yeast was screened using yeast two hybrid assay. A total of 40 colonies were picked up from the four dropout selection and X-gal assay. The list of putative interacting partners of OsIFL protein is given in Table 1. Most of these OsIFL- interacting proteins were the key stress responsive proteins such as, metallothionein, plasma membrane ATPase, palmitoyltransferase TIP1, MSP domain containing protein, ribosome and proton pump interactor. Interaction of OsIFL with these proteins suggest it’s important role in stress tolerance as mediated via these interacting proteins.

**Figure 6:**
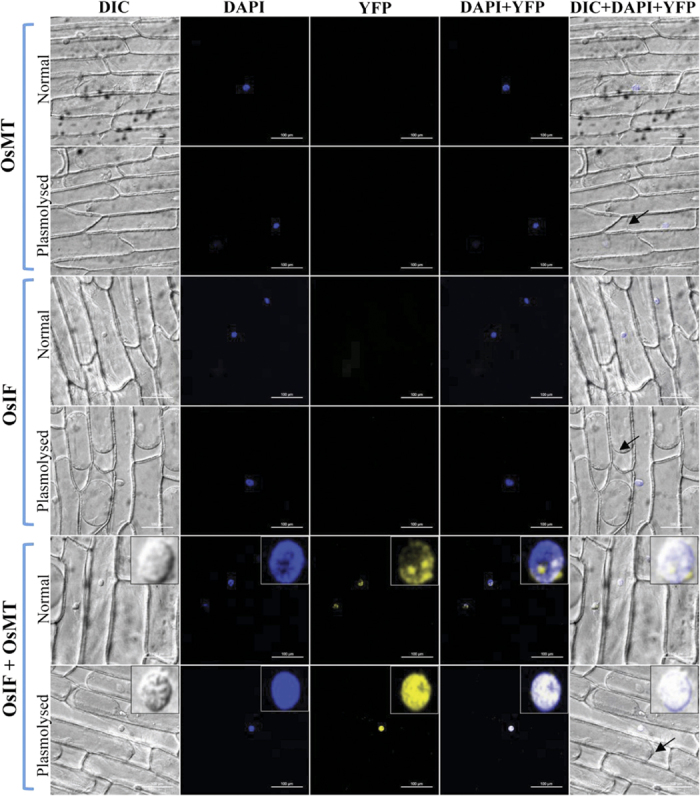
OsIFL interacts with OsMT inside the nucleus and this interaction is enhanced in the presence of salinity stress. BiFC analysis in onion epidermal peel cells expressing full-length OsIFL and OsMT fused to the carboxy (YFPC) or amino (YFPN) terminus of YFP, respectively. Localization of OsMT in non-stress and plasmolysed cells has been shown in first and second rows, respectively. Localization of OsIFL in non-stress and plasmolysed cells has been shown in third and forth rows, respectively; Co-localization of OsMT/OsIFL in non-stress and plasmolysed cells has been shown in fifth and sixth rows, respectively. Yellow fluorescence is visualized only in OsIFL/OsMT co-transformed cells. Arrows in picture shows plasmolysis of cells after 30 min treatment with salt stress. Inset in the lower two panels show enlarged view of the nucleus. Interaction of OsIFL with OsMT is evident clearly in the nucleus (especially in nucleolus) which gets enhanced under salinity stress.

To further confirm the interaction of OsIFL with OsMT, Bi-molecular Fluorescence Complementation (BiFC) assay was performed. *OsIFL* and *OsMT* coding sequence were fused to the C-terminus (YFPC) or N-terminus (YFPN) fragment of yellow fluorescent protein (YFP) to produce YFPC-OsIFL and YFPN-OsMT constructs, respectively. In BiFC assays, onion peel epidermal cells transformed either with only YFPC-OsIFL or only YFPN-OsMT constructs showed DAPI fluorescence but no YFP signals (Fig. 6, upper four panels). However, YFP fluorescence could only be observed in OsIFL and OsMT co-transformed cells. The fluorescence could be seen near the nucleolus which further increased and spread to the whole nucleus after 30 min treatment of onion peel with 200 mM NaCl (Fig. 6, lower two panels). Under both control and stress conditions, yellow fluorescent signal co-localized with the blue fluorescence of DAPI, thus confirming an interaction between OsIFL and OsMT in the nucleus.

## Discussion

Intermediate filaments, well known members of animal cytoskeleton family have recently been reported to be a part of plant cytoskeleton as well^22^,^23^,^24^,^25^,^26^. Evidence presented for *in-situ* localization studies in this report showed OsIFL linked GFP fluorescence in the form of a fibrillar network in the cytoplasm with high abundance at cell margins, supporting that this OsIFL is part of cytoskeleton of the plant cell. Similar pattern of localization of IFs has been reported in pea based on immunofluorescence cytochemistry studies^27^. Since *OsIFL* was found to be induced by salinity, we hypothesize that this protein may have role in improving tolerance towards salinity stress in plants. To evaluate this hypothesis, full-length cDNA of *OsIFL* was ectopically expressed in two lower organisms, *E. coli* (prokaryote) and *S. cerevisiae* (eukaryote). These organisms serve as a good model system as they provide an unparalleled efficiency for identifying and characterizing functionally conserved genes^28^. Recently, some extended coiled-coil proteins were identified in bacterial cells, responsible for maintaining the cell curvature. Crescentin, reported in bacterial cells exhibited most of the peculiar features of IF proteins and termed as IF-like bacterial protein^29^,^30^. Ectopic expression of OsIFL supported the growth of BL21 cells in the presence of different abiotic stressors such as, NaCl (400 mM) and mannitol (400 mM). Since OsIFL was found to be linked to stress response of *E. coli* cells, analysis was extended to non- photosynthetic unicellular eukaryote *S. cerevisiae*. It is believed that some cellular responses towards stress are similar in yeast and plant cells^31^. BY4741 yeast cells ectopically expressing OsIFL also showed better survival under NaCl (1 M) and mannitol (500 mM) stresses. Most of these abiotic stresses lead to production of reactive oxygen species, which in turn, results in oxidative damage to cells. Tolstonog *et al*.^32^ have shown the protective role of vimentin, an IF, in preventing oxidative damage in embryo fibroblast cells. Our findings clearly establish the role of OsIFL in improving tolerance to various abiotic stresses in lower organisms. Using similar approach, we have shown the key role of another stress inducible protein OsCyp2 in improving tolerance towards multiple abiotic stresses in similar set of lower organisms^17^. Recently the usefulness of such an approach has been validated and extended to higher plants such as rice where overexpression of *OsCyp* has been shown to mediate stress response and improve stress tolerance in crop plants via manipulating secondary root growth and hormone homeostasis^33^.

Expression profiling of stress-related marker genes (i.e. *ERD10, LEA5, MT, GST* etc.) has been widely used to study the influence of transgene on plant stress response machinery. Previous studies have indicated that stress marker genes in plants are often involved in different cellular stress responses and induced expression of these genes may contribute to plant survival under abiotic stresses^34^,^35^,^36^,^37^. A vast literature is available showing role of LEA proteins in different abiotic stress, including salinity^34^. Amara *et al*. reviewed role of LEA proteins as stabilizers, hydration buffers, membrane protectants, antioxidants and ion chelators^34^. ERD10 also belongs to LEA 2 family of proteins, whose expression was reported to be induced by a range of abiotic stresses, such as temperature, salinity and drought stress^35^. Expression of glutathione *S*-transferases is also induced by diverse stresses, including salinity^36^. GST helps in maintaining cell redox homeostasis and detoxifying endogenous plant toxins accumulated during oxidative stress^37^. Our results indicate higher expression of these stress-related marker genes in OsIFL transgenic lines than WT plants under salinity stress, suggesting participation of OsIFL in plant stress responses by altering transcript abundance of stress-related genes. Moreover, a higher constitutive expression of these genes in the transgenic tobacco lines even in the absence of any stress also seems to provide an advantage to these plants, thus enabling them to survive under harsh conditions. Higher constitutive expression of stress related transcripts under non-stress conditions appears to be a widely adapted mechanism by salt tolerant wild relatives of plant species^38^.

Yeast two-hybrid experiments also revealed that OsIFL interacts with different types of proteins involved in plant salt stress tolerance, like metallothionein (MT), plasma membrane ATPase, palmitoyl transferases, ribosomes etc. We believe that these interactions are specific and meaningful for abiotic stress tolerance in plants. A large number of studies support active participation of MTs in scavenging of ROS in animals^39^,^40^,^41^. Recent work from our own laboratory has shown that ectopic expression of OsMT enhances tolerance towards multiple abiotic stresses in transgenic tobacco^42^. Xue *et al*.^43^ reported a type 3 MT protein,*GhMT3a*, from cotton (*Gossypium hirsutum*) conferring abiotic stress tolerance to yeast and transgenic tobacco plants by scavenging ROS.

The role of metallothioneins in heavy metal tolerance in plants has been reported by various groups. Its metal binding properties which lead to tolerance to cadmium and zinc homeostasis has also been shown in Arabidopsis^44^. In *Ziziphus jujuba*, up-regulation of *ZjMT* has been reported in response to salt and osmotic stress^45^. Yang M *et al*.^45^ also showed localization of MTs primarily in the cytoplasm and nucleus of cells. Their group documented enhanced salt tolerance by over expression of ZjMT in transgenic Arabidopsis plants at germination stage. Similarly, metallothionein from *Salicornia brachiata* (*SbMT-2*), an extreme halophyte confers tolerance to transgenic tobacco plants towards various abiotic stresses such as, salt, osmotic and heavy metal stress^46^. This study suggested the role of SbMT-2 in cellular homeostasis, H_2_O_2_ detoxification and ROS scavenging. Yang Z *et al*.^47^ reported enhanced drought tolerance in transgenic rice plants overexpressing OsMT1a. They suggested that this enhanced tolerance was not only related to ROS scavenging but also regulated by expression of the zinc finger transcription factors which further led to improved plant tolerance to various stresses. Danielyan *et al*.^48^ has reported co-expression and co-localization of intermediate filament and metallothionein in human, rat skin keratinocytes and fibroblasts. Levadoux *et al*.^49^ have reported association of MT mRNA with the cytoskeleton around the nucleus, which is essential for efficient shuttling of MT into the nucleus during the G1 to S phase transition. In the present study, we have also observed co-localization and interaction of OsMT with cytoskeleton component, OsIFL in nucleus (especially in the nucleolus). In response to salinity stress this interaction was found to be more enhanced and uniformly distributed in the nucleus. Thus, we hypothesize that these IFs may interact with MT and help in maintaining the homeostasis under stress conditions.

As observed for MTs, several environmental factors alter expression levels of the plasma membrane (PM) H^+^-ATPase. Sahu and Shaw^50^ have reported a very high activity of these enzymes even under normal conditions in halophytes and salt-tolerant cultivars than the salt sensitive cultivars. Janicka- Russak^51^ reviewed role of PM H^+^ATPase in different abiotic stress tolerance. In plants, PM H^+^-ATPase regulates nutrient uptake, intracellular pH, stomatal movements and cell growth. By regulating all these physiological processes, plasma membrane proton pump helps in stress adaptation of plants. Interaction of PM H^+^-ATPase with IFs may help in regulating the stomatal movements and cell growth as a stress response. Heterologous expression of Palmitoyltransferase TIP1 from *Glycine max* (*GmTIP1;1*) confers salt tolerance to the yeast cells^52^. Since TIP1 is a putative interacting partner of OsIFL, we propose that IFs might be a part of TIP1 salt stress response machinery in plants.

Our results corroborated with the previous study by Traub *et al*.^53^ which reported interaction of IFs with single ribosome. Their studies reported IFs as a part of storage machinery of ribonucleoprotein particles and non-translating ribosomes, in the cytoplasm of animal cells. In our study, OsIFL has been found to be localized in nucleolus where rRNA is assembled with ribosomal proteins to form nearly completed pre-ribosomal subunits, ready for export to the cytoplasm. However, detailed investigations are required to establish the role of OsIFL in ribosomal assembly.

For the purpose of studying effect of expression of transgene - *OsIFL* in model plant tobacco, we analyzed various physiological parameters in the transgenic plants under salinity stress. For this purpose, we carried out this assessment of stress tolerance in leaf discs of the transgenic plants (to comment on cellular tolerance), plants of T_1_ generation as well as that of T_2_ generation (to comment on effects brought in by the stable integration of OsIFL at whole plant level). Leaf disc assay has been an easy and quick way to assess the tolerance of plants towards abiotic stresses in various studies^54^. In this test, the transgenic plants showed less chlorophyll loss than the WT plants under salinity stress, indicating improved capacity of the former to maintain the levels of chlorophyll (Fig. 4a,b). In the presence of 150 mM salt stress, transgenic OsIFL lines showed better performance in terms of different physiological parameters such as, relative water content, K^+^/Na^+^ ratio, chlorophyll content, electrolyte leakage etc. (Fig. 4c–g). Subsequently, T_2_ generation seeds of the transgenic lines and WT plants were germinated on MS media in the presence of salt. Almost 100% germination efficiency of transgenic seeds under these conditions suggested role of OsIFL in conferring tolerance towards salt stress at the seed germination stage (Fig. 4h).

**Figure 7:**
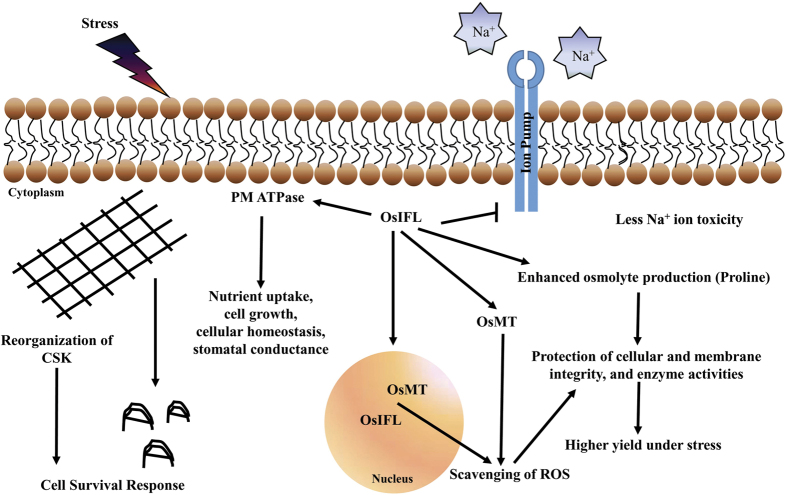
Possible mechanism of OsIFL involvement in plant stress response. Based on the present study, it is likely to believe that OsIFL may have versatile functions in plant cell. For example, OsIFL decrease ion import in cells and thus prevent ion toxicity under salinity stress. It interacts with metallothionein and play important role in ROS scavenging. OsIFL also interacts with plasma membrane ATPase (PM ATPase) and regulate nutrient uptake and cell growth. It also promotes osmolyte production to maintain cellular homeostasis. It is also reported that there is re-organization of cytoskeleton (CSK) in response to stress, which may contribute indirectly to cell survival.

Osmolytes such as proline, helps in protection of cellular structures and scavenging of ROS^55^,^56^. These compounds assist in maintaining integrity of the membranes and keep the photosynthetic system functioning^56^. OsIFL transgenic plants also showed higher accumulation of proline than WT plants, which might be a reason of better retention of chlorophyll content in these plants under stress (Fig. 4g). These results strongly indicate that OsIFL transgenic plants have enhanced or better stress tolerance than their WT counterparts. All these observations led us to propose the various possibilities of how IF might contribute to stress tolerance. IFs might support cell survival by reorganizing themselves under stress, helping in ROS scavenging by interacting with MTs and other stress proteins. IFs may also influence ion accumulation or transportation by interacting with some ion transporters i.e. proton pump interactor and PM H^+^ATPase. By regulating osmolyte production, it might also help in maintaining cellular integrity and physiological processes like photosynthesis, which in turn, leads to better performance and enhanced stress tolerance of transgenic plants (Fig. 7).

## Conclusions

This study provides the first insight into the role of intermediate filaments in plants salinity stress response. We hypothesize a conserved role of OsIFL in abiotic stress tolerance across genera, i.e. plants as well as lower organisms, as its over expression provides tolerance towards different abiotic stresses. OsIFL interacts with several other stress related proteins, such as MT, PM-ATPase, palmitoyl transferases, ribosomes etc. We found interaction of OsIFL with metallothionein in the nucleus (especially the nucleolus) of cells. This interaction increased further throughout the nucleus in response to salinity stress. We hypothesize that OsIFL is a key component of stress responsive machinery and through interaction with other specific proteins, OsIFL regulates cellular homeostasis and provide survival advantage to cells of diverse organisms.

## Methods

### Sequence analysis and cloning of rice intermediate filament (*OsIFL*)

Protein and gene sequences of *OsIFL*, LOC_Os01g18840 were retrieved from TIGR database (rice genome annotation project ver. 7). SMART (www.smart.embl-heidelberg.de) and pfam (http://pfam.sanger.ac.uk/) tools were used for domain analysis of proteins. Secondary structure prediction was carried out using PSIPRED, protein structure prediction server (http://bioinf.cs.ucl.ac.uk/psipred/). Coiled coils were predicted with the alogrithm called multicoil2 (http://groups.csail.mit.edu/cb/multicoil/cgi-bin/multicoil.cgi). For the amplification of *OsIFL* cDNA, forward and reverse primer pair, 5′-ATGCCGATTTCAGAGAGGGC-3′ and 5′-TCATCGCTTCGACAGCATAG-3′, were designed and synthesized according to the *OsIFL* sequence, LOC_Os01g18840. The total RNA was extracted from shoot tissues of 7 day old rice seedlings using TRI reagent (Sigma, USA). First-strand cDNA synthesis was carried out using RevertAid*™* cDNA synthesis kit (Fermentas Life Sciences, USA) as per manufacturer’s instructions. This cDNA was then used as a template for the polymerase chain reaction (PCR) using Phusion Taq polymerase (NEB, USA). PCR products were analyzed on agarose gel and purified with Qiagen gel extraction column (Qiagen, Germany) and cloned into TopoTA vector. Cloned fragment was sequenced and confirmed to be *OsIFL*. The gene was subsequently cloned into bacterial (pET28a), yeast (pYES2) or plant (pCAMBIA1304) expression vectors for expression of OsIFL protein in *E. coli* cells, yeast and tobacco, respectively.

### Subcellular localization of OsIFL

The complete ORF (Open Reading Frame) of *OsIFL* was cloned into the pCAMBIA1304 vector at *NcoI* and *SpeI* site to create a GFP fusion construct (pCAMBIA1304-OsIFL-GFP). pCAMBIA1304-OsIFL-GFP construct (along with empty vector as control) were transformed in onion peel epidermal cells using particle bombardment as done previously^18^. The GFP fluorescence was visualized after 24 h incubation (at 28 °C in dark) using a confocal microscope. The nuclei were stained with DAPI.

### Ectopic expression of OsIFL in *E. coli* and analysis of its growth in presence of salinity and osmotic stresses

*E. coli* BL21 (DE3) cells were transformed with pET28a vector containing *OsIFL* cDNA or with empty pET28a vector and grown in 5 ml culture at 37 °C, induced with 0.3 mM IPTG and further grown for 2 h (OD_600_ = 0.5) at 22 ± 1 °C for induction of OsIFL protein. Finally, equal inoculum, determined by turbidometric method^57^ was used for serial dilution onto the solid LB media supplemented with either 400 mM NaCl or 400 mannitol, both containing 0.3 mM IPTG, under kanamycin selection (50 μg/ml) and incubated at 22 ± 1 °C for 6 h for induction of OsIFL protein followed by incubation at 37 ± 1 °C for 72 h, after which the plates were photographed.

### Transformation of *S. cerevisiae* with OsIFL and assessment of their growth in the presence of stress

Full length *OsIFL* cDNA was cloned in the yeast expression vector pYES2, downstream to the galactose inducible promoter. *S. cerevisiae* BY4741 (*Mat a; his3Δ1 leu2Δ0 met15Δ0 ura3Δ0*) cells were transformed with pYES2 and pYES2OsIFL construct and the transformants were picked up by *ura* prototrophy. For checking the growth profile of these yeast cells under stress, transformants were first grown in liquid YPG containing 2% galactose, at 30 °C for 48 h (till saturation). Thereafter, equal inoculum, as determined by the turbidometric method, was allowed to grow at 30 °C onto solid YPG media in presence of either NaCl (1 M) or mannitol (500 mM) and the colonies were photographed after 72 h. For preparing the growth curve, inoculum was added to liquid YPG media to obtain initial OD_600_ = 0.3, growth curve was monitored in presence of 1 M NaCl or 1M Mannitol for 24 h at 30 °C.

### Construction of plant expression vector and transformation of tobacco

To express OsIFL in tobacco (*Nicotiana tabacum* var. petit havana), the full length cDNA was cloned at *Nco*I and *Spe*I sites of plant expression vector pCAMBIA1304, which was flanked by CaMV35S promoter and the poly-adenylation signal to create pCAMBIA1304OsIFL. The resultant recombinant vector pCAMBIA1304OsIFLwas transformed into *Agrobacterium tumefaciens* strain GV1304 by a freeze–thaw method. Single colony of *Agrobacterium* strain was grown at 28 °C in 50 ml of liquid LB medium containing 50 mg/l gentamicin, 25 mg/l rifampicin and 50 mg/l kanamycin to post-log phase. Then, the bacterial suspension was pelleted at 3000 rpm for 10 min and the cells were resuspended in MS medium to maintain the O.D_600nm_ ~0.3. This Agrobacterium suspension containing pCAMBIA1304OsIFLwas used for co-cultivation of leaf discs of 3–4 week old tobacco plants grown under sterile condition in culture room as described previously^54^. The putative T_0_ transformants were screened using tissue PCR, Southern and Western blot analyses.

### Molecular confirmation of transgenic tobacco by tissue PCR, RT-PCR, Southern blotting and western blotting

Tissue PCR was done using Red Extract-N-Amp kit (Sigma-Aldrich, India), as per the manufacturer’s instructions. The gene specific forward primer 5′-ATGGCGCCGATTTCAGAGAGGGC-3′ and vector specific reverse primer 5′-CTGGAGTTGTCCCAATTCTTG-3′ were used to amplify 849 bp fragment of the *OsIFL* gene. PCR was performed with an initial denaturation step at 95 °C for 5 min; followed by 35 cycles at 95 °C for 1 min, 58 °C for 1 min, and 72 °C for 1 min; and a final extension at 72 °C for 10 min. The PCR products were separated by electrophoresis on a 1% (w/v) agarose gel.

The expression of OsIFL mRNA was analyzed by reverse transcriptase PCR (RT-PCR). Total RNA was extracted from leaf tissue using TRI reagent (Sigma, USA), treated with RNase-free DNase I and reverse transcribed using MMLV Reverse Transcriptase RNaseH (Toyobo, Japan). Gene specific primers were used for the RT-PCR analysis to amplify an 807 bp product, and actin gene-specific primers were used as the internal control.

For Southern blotting, 30 μg of genomic DNA was digested with *Nco*I and *Spe*I to detect the presence of *OsIFL*. The digested products were purified and fractionated on 0.8% agarose gel, subsequently transferred to an Amersham Hybond N^+^ nylon membrane (GE Healthcare Life Sciences) by capillary transfer. An 807 bp [αP^32^] ATP labeled fragment of *OsIFL* cDNA was amplified using the gene specific primers and DecaLabel kit according to manufacturer’s instructions (Fermentas, Germany). The UV-cross linked membrane was pre-hybridized at 65 °C for 3 hr in buffer containing 5x SSC, 5x denhardt reagent (0.5% Ficoll, 0.5% PVP, 0.5% BSA), 0.1% SDS, 100 μg/ml denatured salmon sperm DNA and 10% dextran sulphate and hybridized for 16 h with a cDNA probe radiolabeled with [^32^P] ATP. After hybridization, the membrane was washed twice with 2x SSC for 5 min, 2x SSC + 0.1% SDS for 10 min and 0.1% SDS for 5 min. Washed membrane was exposed to phosphorimager plate (Amersham Pharmacia Biotech, England) for 15 h and scanned to visualize the hybridized band.

Expression of OsIFL protein in transgenic tobacco plants was analyzed by Western blot analysis. For this purpose, leaves from WT and transgenic lines were ground in liquid nitrogen and the total proteins were extracted using Zivy’s buffer as described previously^18^ and quantified. A 10-μg sample of total protein was mixed with loading buffer, boiled for 3 min, and size fractionated through electrophoresis in a 12.5% SDS polyacrylamide gel. Separated proteins were transferred to Hybond-C^+^ membrane (Amersham Pharmacia Biotech, England) using Mini Transblot Electrophoretic cell (Biorad, USA). Electroblotting buffer consisted of 150 mM glycine, 20 mM Tris and 20% methanol (pH 8.0). The blot was rinsed briefly in phosphate buffered saline or PBS [137 mM NaCl, 2.7 mM KCl, 4.3 mM Na_2_HPO_4_.7H_2_O, 1.4 mM KH_2_PO_4_ (pH 7.3)] and then incubated in blocking solution (5% non-fat dried milk dissolved in PBS). Blot was then washed with PBS and thereafter, incubated with anti-IF antibodies (custom synthesized by Bangalore Genei, India). Blot was further incubated in Alkaline-Phosphatase conjugated secondary antibodies. The protein-antibody complex was developed in 5-bromo-4-chloro-3-indolyl phosphate (BCIP)/nitrobluetetrazolium (NBT) containing solution (Sigma-Aldrich, USA).

### Analysis of transgenic tobacco plants for abiotic stress tolerance

#### Leaf disc assay

Leaf discs were excised from healthy and fully expanded tobacco leaves of 8-week-old WT and T_0_ transgenic lines (L2 and L3) using a cork borer. Leaf discs were floated in 10 ml solution of half MS alone (as control) or containing 150 mM NaCl (for salinity stress) in petriplates. These petriplates were kept in plant growth chambers maintained at 28 ± 1 °C. After 48h, these leaf discs were photographed and used for measuring total chlorophyll content spectrophotometrically, as described by Arnon *et al*.^58^.

#### Seed germination and growth under stress conditions

T_1_ generation seeds of OsIFL expressing transgenic tobacco line, along with WT, were subjected to salinity stress. For this purpose, sterilized seeds from transgenic line along with WT were sown on the freshly prepared half MS agar plates. After 7 day, seedlings were transferred to vermiculite. After 30 days, one set of WT plants and two transgenic lines (L2 and L3) were subjected to salinity stress by watering them with a solution of 150 mM NaCl and the other set was watered normally as controls. The growth of plants was monitored for three weeks. Various physiological parameters e.g. relative water content, proline accumulation in μg/gm fresh weight, K^+^/Na^+^ ratio and electrolyte leakage were measured. At the next generation (T_2_), the seed germination assay under salinity was carried out. For this, equal number of sterilized seeds of both transgenic lines along with WT was sown on freshly prepared half MS agar plates or the media supplemented with 150 mM NaCl. After 15 days of sowing, images were taken and analysed.

#### Relative water content

Whole seedlings of similar physiological age were used for the determination of the relative water content (RWC = FW − DW/TW − DW, where FW: fresh weight, DW: dry weight, and TW: turgid weight). The fresh weight of seedlings was determined, and then seedlings were kept in distilled water for 4 h, at room temperature (25 °C), before turgid weight of seedlings was determined. Seedlings were then dried for 48 h at 65 °C to determine their dry weight.

#### Estimation of K^+^ and Na^+^

For the determination of endogenous Na^+^ and K^+^ content, 100 mg of leaf tissue (control or stressed) was taken and digested twice in 0.1% HNO_3_ for 30 min and 10 min in boiling water bath, followed by filtering the extract through Whatman no. 40 (ashless). Ions were then extracted in distilled H_2_O by boiling for 10 min. The filtered extract thus obtained, was used to measure specific ions with a flame photometer. Experiment was repeated 3 times, and standard errors were calculated (n = 3).

#### Electrolyte leakage measurement

Analysis of electrolyte leakage was carried out as done previously^18^. Essentially, seedlings were harvested from both stressed as well as unstressed (control) samples, then quickly washed with distilled water to remove the surface adhering ions leached during the salt treatment followed by small incisions in the tissue samples. About 100 mg tissue was immediately dipped into 20 ml of de-ionized water. After incubating the seedlings at 37 °C for 2 h, electrical conductivity (E_1_) of the immersion solution was measured using a conductivity meter (EleinsInc, India). To determine total conductivity (E_2_), the seedlings, with immersion solution (effusate), were autoclaved for 15 min at 121 °C and the conductivity of the effusate was measured after cooling it to room temperature. For each time point in the above measurement, the experiment was repeated at least three times. For each treatment, standard deviation was also calculated. Relative electrical conductivity was calculated by the formula:


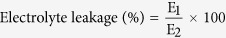


#### Determination of proline content

Proline was extracted from 0.1 g freeze-dried leaf samples and spectrophotometrically determined using the acid ninhydrin method published by Bates *et al*.^59^.

#### qRT-PCR based expression analysis for stress related genes

For the evaluation of transcript levels of various stress related genes (GST, ERD, LEA and MT), 4-week-old tobacco plants were treated with 200 mM NaCl for 24 h, while seedlings treated with water served as controls. Total RNA was extracted using Trizol reagent (sigma) and treated with RNase-free DNase I and reverse transcribed using M-MLV Reverse Transcriptase RNaseH (Toyobo, Japan). The qRT-PCR analysis was performed as described earlier^18^. Averaged expression of tobacco ubiquitin gene (*NiUb*) was used as reference for normalization. Sequence of primers used in qRT-PCR were as follows: NtUb-F: TCCAGGACAAGGAGGGTAT, NtUb-R: CATCAACAACAGGCAACCTAG; NtMT-F: CCATGAAACTGACCATCTCC, NtMT-R: TTACAGCCCAAATCAGCTTC; NtGST-F: CCCCTAGTTTGCTCCCTTCT, NtGST-R: TTCTTAGCTGCCTCCTGCTC; NtLEA5-F: TTGAATCTGGGGTTTTGGTT, NtLEA5-R: GGAAGCATTGACGAGCTAGG; NtERD10-F: AACGTGGAGGCTACAGATCG, NtERD10-R: GTTCCTCTTGGGCATGAGTT.

#### Screening of rice cDNA library for identification of interacting partners of intermediate filaments

Rice salt stress related cDNA library cloned in pAD-GAL4-2.1 vector and transformed in AH109 yeast strain^60^ was used for this experiment. Competent cells prepared with these AH109 cells were subsequently transformed with pBD-GAL4OsIFL construct. Transformed cells were plated over double dropout media (SD-Leu/-Trp). Obtained colonies were further streaked onto the triple dropout media (SD-Leu/-Trp/-His) and four dropout media (SD-Leu/-Trp/-His/-Ade). Colonies with putative interacting partners were further confirmed by filter lift assay. Plasmids were isolated from these positive colonies and transformed into DH5α bacterial cells. Plasmids from these bacterial colonies were isolated and sequenced using pGAD vector specific primers. Gene sequences which were ‘in frame’ with activation domain of pGAD vector were then analyzed by Expasy translation tool (http://web.expasy.org/translate). The largest ORF was selected and used for identification of putative interacting partner through Blastn, Blastx and Blastp tools of TIGR database.

#### Protein-Protein interaction analysis using BiFC

To reconfirm the protein-protein interaction *in planta*, BiFC assay was carried out in onion peel epidermal cells. The full length coding sequences of *OsMT* and *OsIFL* were cloned ‘in frame’ with the N-terminus and C-terminus region of YFP using pSAT-EYFP-N and pSAT-EYFP-C vector, respectively. Constructs were confirmed by restriction digestion as well as sequencing. Onion peel epidermal cells were transiently co-transformed either with both constructs or with one of the constructs, as fluorescence control, using particle bombardment. Interaction in the onion peel was monitored after 24 h of culture on MS medium at 28 °C in dark. For salinity treatment, the peels were kept in 200 mM NaCl solution for 30 min. The peels were also stained with DAPI for visualization of nuclei. Transformed cells, both normal and plasmolyzed, were visualized for YFP fluorescence and DAPI using confocal microscope.

### Statistical analysis

All experiments were repeated thrice and standard deviations (s.d.) were recorded. Statistically significant observations were presented by an asterisk in graphs, as determined by Student’s t-test (p < 0.05).

## Additional Information

**How to cite this article**: Soda, N. *et al*. Evidence for nuclear interaction of a cytoskeleton protein (OsIFL) with metallothionein and its role in salinity stress tolerance. *Sci. Rep*. **6**, 34762; doi: 10.1038/srep34762 (2016).

## Supplementary Material

Supplementary Information

## Figures and Tables

**Table 1:** List of putative interacting partners of OsIFL protein.

Protein	No of clones showing the specific interaction	Locus ID
Metallothionein	3	LOC_Os12g38270
Plasma Membrane ATPase	2	LOC_Os04g56160
Palmitoyltransferase TIP1	2	LOC_Os02g09130
MSP Domain Containing Protein	1	LOC_Os02g42940
Ribosome	2	LOC_Os02g09590
Expressed protein	3	LOC_Os01g70400
Uncharacterized ACR, YggU family COG1872 containing protein	2	LOC_Os07 g19470
Proton Pump Interactor	1	LOC_Os08g30060
