# Supplementary material for: Evidence for nuclear interaction of a cytoskeleton protein (OsIFL) with metallothionein and its role in salinity stress tolerance
Source: Sci Rep. 2016 Oct 6;6:34762. doi: 10.1038/srep34762 (PMC5052524; doi:10.1038/srep34762)
Supplement: Supplementary Information [file srep34762-s1.pdf]

Supplementary information

Evidence for nuclear interaction of a cytoskeleton protein (OsIFL) with metallothionein and its role in salinity stress tolerance

Neelam Soda[1], Ashutosh Sharan[1], Brijesh K. Gupta[2], Sneh L Singla-Pareek[2], Ashwani Pareek[1*]

[1]Stress Physiology and Molecular Biology Laboratory, School of Life Sciences, Jawaharlal Nehru University, New Delhi 110067, India,

[2]Plant Stress Biology, International Centre for Genetic Engineering and Biotechnology, Aruna Asaf Ali Road, New Delhi 110067, India

*To whom correspondence should be addressed:

Ashwani Pareek,

Stress Physiology and Molecular Biology Laboratory,

School of Life Sciences, Jawaharlal Nehru University,

New Delhi 110067, India

Phone: +91-11-26704504 (O);

Fax No.: +91 11 26742558

E-mail: ashwanip@mail.jnu.ac.in

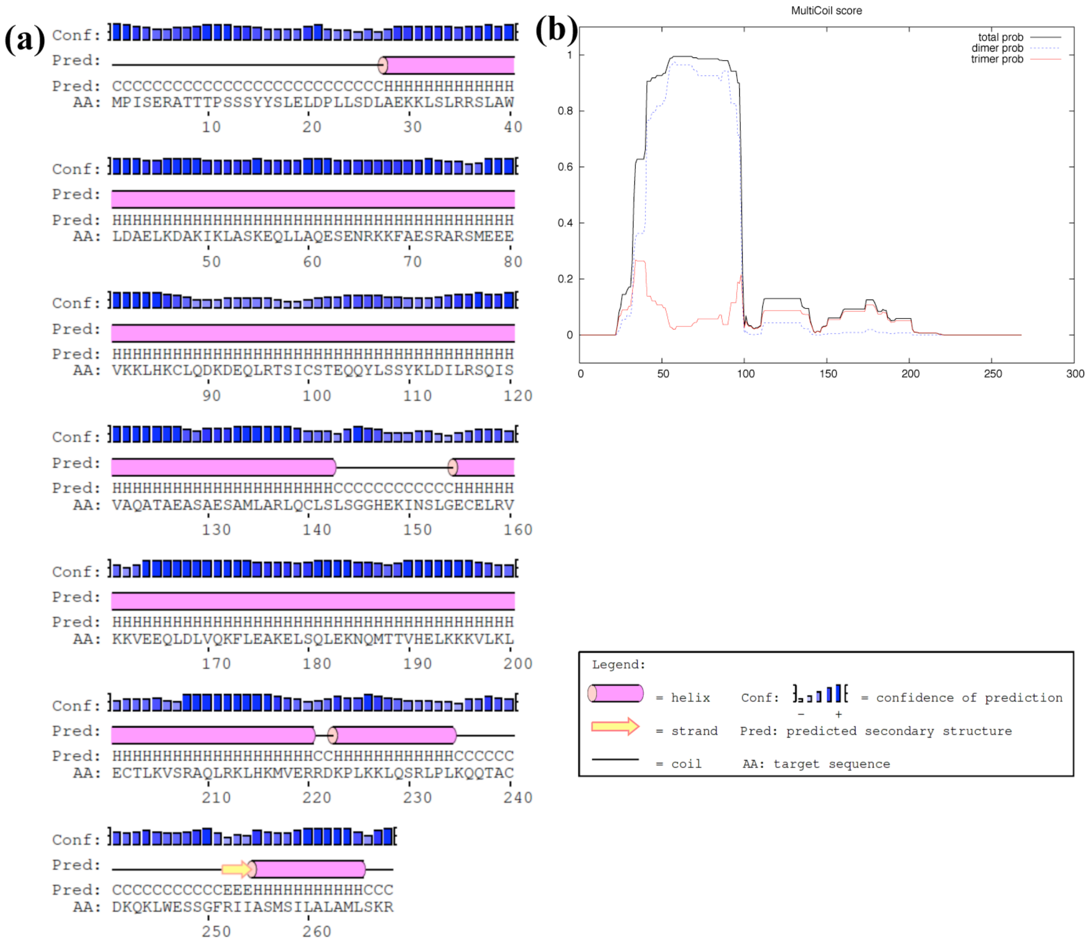

Supp. Figure 1: OsIFL is a coiled coil forming protein. (a) Secondary structure of OsIFL protein using PSIPRED tool, showing presence of five alpha helices; (b) Probability graph made by using Multicoil tool shows high probability of coil formation at N-terminal of OsIFL.

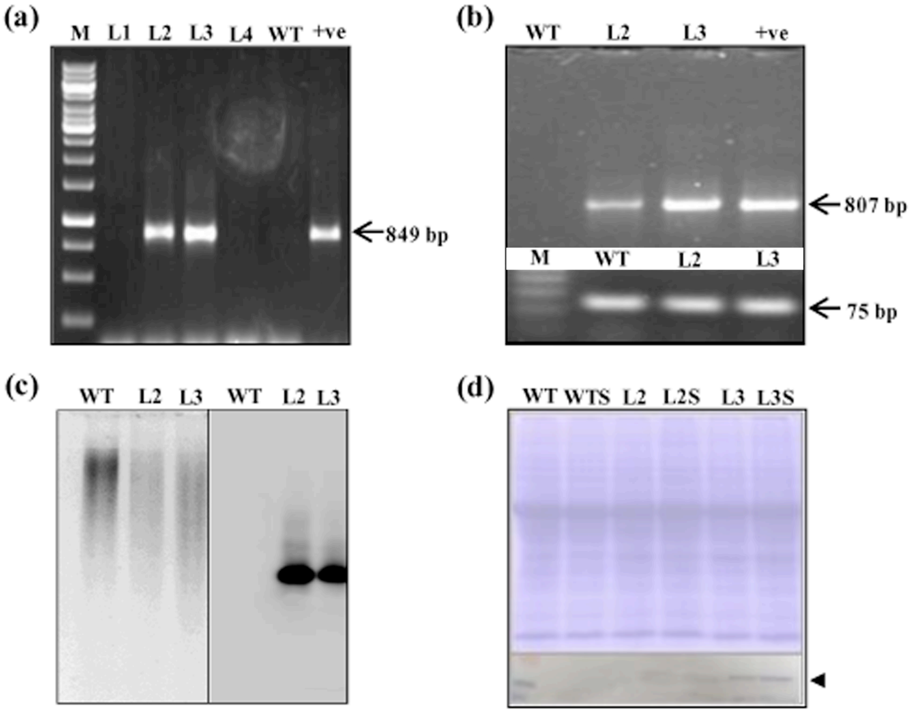

Supp. Figure 2: Molecular confirmation of transgenic tobacco plants. (a) EtBr gel showing screening of putative transgenic tobacco plants transformed with *OsIFL*. Positive transformants were selected using a combination of gene specific (*OsIFLNco*-F) and vector specific (pCAMBIA-R) primer pair for the presence of 849 bp amplicon which can be seen in line L2 and L3 only (marked by arrow). Genomic DNA from wild type (WT) plant was taken as negative control and pCAMBIA1304*OsIFL* plasmid was taken as positive control; (b) EtBr gel showing RT-PCR analysis of putative transgenic lines with *OsIFL* gene specific primers, an amplification of 807 bp can be seen only in L2 and L3 lines. No band was detected in WT tobacco plants. Lower panel of EtBr gel showing RT-PCR analysis with tobacco actin gene specific primers, a 75 bp tobacco actin gene was amplified from cDNA of WT plants lane 2 and both transgenic lines lane 3, 4; lane 1 has 50 bp marker; (c) 30 μg genomic DNA from both WT tobacco and transgenic tobacco lines (L2 and L3) overexpressing *OsIFL*was digested with *Nco*I and *Spe*I (left panel). Southern blot showing fallout in both the transgenic lines. No fallout was detected in WT tobacco plants (right panel). Radiolabeled *OsIFL* gene was used as probe; (d) Western blot analysis of transgenic lines. Coomassie stained SDS PAGE from wild type tobacco plants as well as *OsIFL* transgenic lines L2 and L3 and blotted on the nylon membrane. Western blotting was carried out with anti-OsIFL antibodies which cross-react to give a specific band of ~30 kDa (shown by arrow). WT, L2 and L3 represent the sample from un-stressed plants while WTS, L2S and L3S represents the samples from salinity stressed plants.
